# Supplementary material for: Training confounder-free deep learning models for medical applications
Source: Nat Commun. 2020 Nov 26;11:6010. doi: 10.1038/s41467-020-19784-9 (PMC7691500; doi:10.1038/s41467-020-19784-9)
Supplement: Supplementary file 1 — Supplementary Information [file 41467_2020_19784_MOESM1_ESM.pdf]

Supplementary Information

Training Confounder-Free Deep Learning Models for Medical Applications

Qingyu Zhao†, Ehsan Adeli†, Kilian M. Pohl*

†Equal contribution lead author; *Corresponding Author

{qingyuz,eadeli,kilian.pohl}@stanford.edu

Supplementary Methods and Notes

Synthetic Experiments. We generated a synthetic dataset comprised of two groups of data, each containing 512 images of resolution 32×32 pixels. Each image was generated by 4 Gaussians (see Supplementary Figure 1(a)), the magnitude of which was controlled by σ and $\mathbf{c}$. For each image from Group 1, we sampled σ and $\mathbf{c}$ from a uniform Gaussian distribution $\mathcal{U}(1, 4)$ while we generated images of Group 2 with stronger intensities by sampling both variables from $\mathcal{U}(3, 6)$ (Supplementary Figure 1(b-c)). Gaussian noise was added to the images with standard deviation 0.01. Now we assume the difference in σ between the two groups is associated with the true discriminative cues that should be learned by a classifier, whereas $\mathbf{c}$ is a confounder variable. In other words, an unbiased model should predict the group label purely based on the two diagonal Gaussians and not dependent on the two off-diagonal ones. Due to the overlap in the sampling range of σ, the optimal classification accuracy for the two groups was 90% (Supplementary Figure 1(b)). To show that the CF-Net can result in such models by controlling for $\mathbf{c}$, we trained it on the whole dataset of 1,024 images with binary labels $\mathbf{y}$ and the corresponding confounder values $\mathbf{c}$ as the input.

Network Architecture. $\mathbb{FE}$ produced 32 features by 3 convolutional stacks, where each stack was composed of a 2×2 convolution, ReLU, and max-pooling layer. Both the $\mathbb{CP}$ and $\mathbb{P}$ networks had one hidden layer of dimension 16 with *tanh* as the non-linear activation function. Two implementations of CF-Net were tested. The first implementation only employed $\mathbb{CP}$ to Group 1 (modeling conditional dependency between $\mathbf{F}$ and $\mathbf{c}$ w.r.t $\mathbf{y} = 0$), and the second employed $\mathbb{CP}$ to all training samples (modeling full dependency between $\mathbf{F}$ and $\mathbf{c}$, without conditioning on $\mathbf{y}$).

Evaluation. Beyond recording the classification accuracy after each iteration, we quantified the conditional dependency between the learned features $\mathbf{F}$ and the confounder $\mathbf{c}$ by measuring their squared distance correlation ($dcor^2$) [9] and mutual information (MI) [1]. By separately computing those metrics for each group, we accounted for the conditional dependency between $\mathbf{F}$ and $\mathbf{c}$ (conditioned on $\mathbf{y} = 0$ or $\mathbf{y} = 1$). We not only computed these scores for the baseline ConvNet and the proposed CF-Net but also for implementations of CF-Net replacing the adversarial loss function (Eq. 2 in main article) with the losses proposed in the state-of-the-art invariance learning

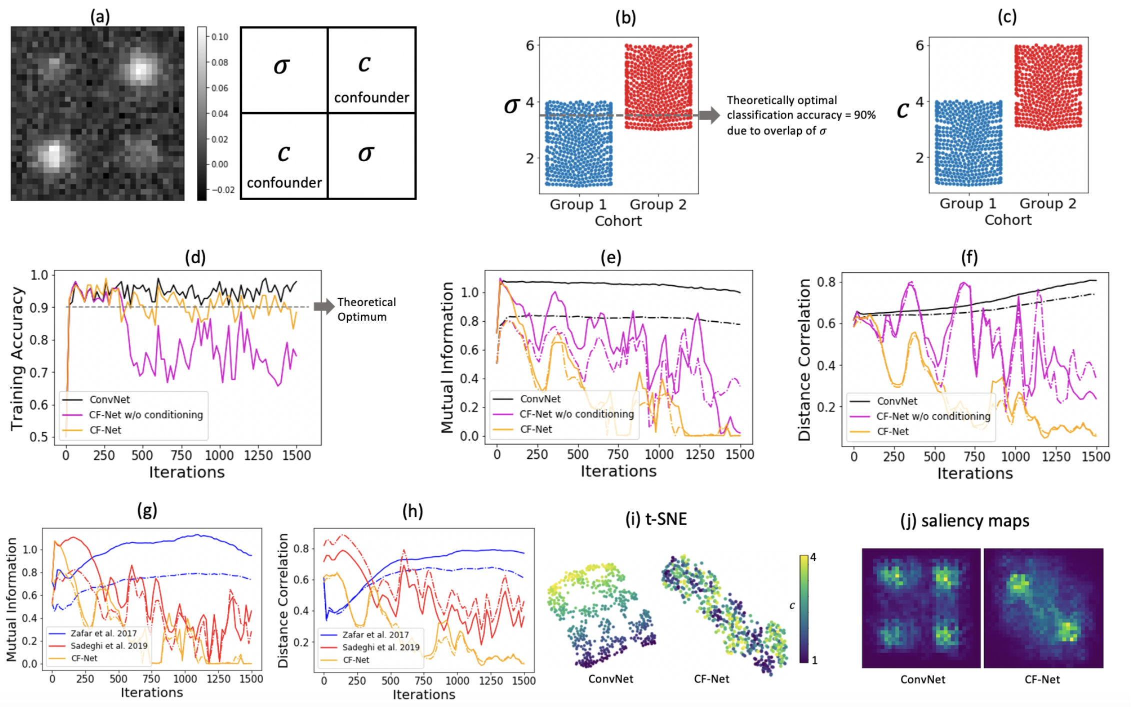

Supplementary Figure 1: (a) An example synthetic image, in which the 4 Gaussians were controlled by their magnitude σ and $\mathbf{c}$; (b,c) Sampling of σ and the confounder $\mathbf{c}$ across the 1024 synthetic images; (d) Training accuracy of ConvNet and CF-Net with respect to number of iterations; (e,f) Distance correlation (*dcor*) and mutual information (MI) measured between the learned features and the confounder $\mathbf{c}$ within each group (solid curves for Group 1, dashed curves for Group 2); (g,h) *dcor* and MI measured for CF-Net and two other invariant feature approaches; (i) t-SNE visualization of the learned feature spaces; (j) Average saliency maps derived for the ConvNet and CF-Net.

frameworks by Sadeghi et al. [7] and Zafar et al. [12]. Note, the majority of invariant or unbiased feature learning approaches (see [1] for review) focus on binary bias variables so that they cannot be applied to this experiment.

Beyond this quantitative assessment, we visualized the impact of $\mathbf{c}$ on the high-dimensional feature space via t-SNE [4], which projected the features $\mathbf{F}$ of each training sample into 2D and color-coded the projection point with the value of $\mathbf{c}$. Lastly, we computed the "saliency map" for each training sample [3], which provided a voxel-wise measure quantifying the importance of each voxel in the final prediction. We visualized the average saliency maps over all 1,024 images.

Results. The training accuracy of the baseline ConvNet went beyond the theoretical optimum of 90% (Supplementary Figure 1(d)), indicating that the model falsely leveraged the off-diagonal Gaussians (linked to $\mathbf{c}$) in the image for prediction. This observation was supported by the following three findings: 1) the relatively high statistical dependency between $\mathbf{F}$ and $\mathbf{c}$ according to MI and *dcor* metrics (Supplementary Figure 1(e-f)); 2) a strong correlation between $\mathbf{c}$ and the projections of the features in the 2D space (Supplementary Figure 1(i)); 3) all fours Gaussians had high

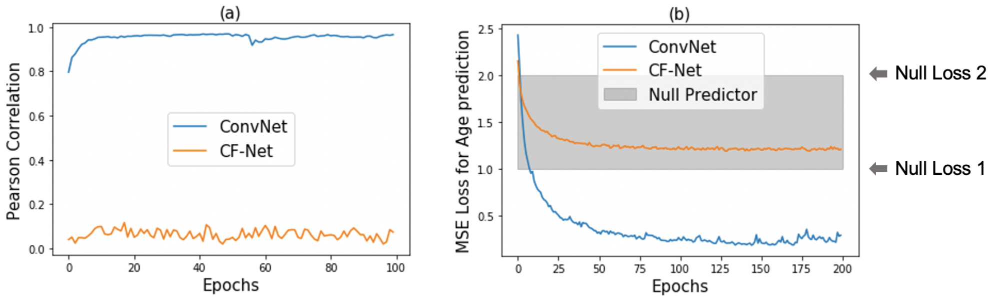

Supplementary Figure 2: HIV experiment: After each training run of the 5-fold cross-validation, $\mathbb{CP}$ of ConvNet and CF-Net was further trained on the features extracted by the model to predict age. Only the features extracted by ConvNet were predictive of age indicated by the (a) high correlation and (b) low MSE loss recorded on the testing folds.

importance for the prediction according to the saliency map (Supplementary Figure 1(j)). Only the implementation of CF-Net with $\mathbf{y}$-conditioning matched the training accuracy of 90%, indicating that the model only leveraged the two diagonal Gaussians for prediction. This was supported by reduced *dcor* and MI scores, t-SNE visualization, and the saliency map. Despite that the modelling of confounding effect was specifically conditioned on Group 1, the conditional dependency between $\mathbf{F}$ and $\mathbf{c}$ was removed in both groups (solid and dashed curves in Supplementary Figure 1(e-f)) by CF-Net.

Note, CF-Net without $\mathbf{y}$ conditioning (that applied $\mathbb{CP}$ to all samples) achieved suboptimal results. This implementation essentially aimed to remove full dependence between $\mathbf{F}$ and $\mathbf{c}$ regardless of their indirect link caused by the intrinsic correlation between $\mathbf{y}$ and $\mathbf{c}$. In other words, $\mathbb{CP}$ not only removed the direct association between $\mathbf{F}$ and $\mathbf{c}$ (link ①), but also minimized the dependency between $\mathbf{F}$ and $\mathbf{y}$ to reduce the indirect association between $\mathbf{F}$ and $\mathbf{c}$ (link ② & ③). This contradicted the objective of $\mathbb{P}$ that aimed to maximize the dependency between $\mathbf{F}$ and $\mathbf{y}$. Therefore, the contradictory objectives simultaneously resulted in comprised prediction accuracy (Supplementary Figure 1(d)), *dcor* (Supplementary Figure 1(e)), and MI results (Supplementary Figure 1(f)).

Lastly, the adversarial loss of CF-Net outperformed loss functions from other state-of-the-art methods in deriving high-dimensional features impartial to confounders (Supplementary Figure 1(g-h)). This was due to the fact that our adversarial objective was specifically designed for pursuing statistical mean independence between the features and confounder. We refer reader to our technical report [1] for an extensive discussion on the theoretical properties of our method, statistical analysis on the high-dimensional features, and comparison with other baseline methods.

Additional Results on HIV Diagnosis from MRIs. This subsection outlines additional results and analysis of model hyperparamerters for the HIV diagnosis experiment.

<u>Age prediction by $\mathbb{CP}$.</u> After fully training CF-Net and ConvNet (i.e., the training loss in each run of the 5-fold cross-validation converged), we measured the accuracy of $\mathbb{CP}$ in predicting confounding

Supplementary Table 1: Top: Classification of HIV diagnosis: Balanced accuracy, precision, recall, and F_1-score of HIV diagnosis prediction. Best results in each column are typeset in bold; Bottom: Uncorrected p-value of DeLong's test associated with the improvement of each method over the ConvNet baseline.

	Whole Cohort				**c**-independent subset		**c**-independent Young		**c**-independent Old	
**Method**	**BAcc (%)**	**Pre (%)**	**Rec (%)**	**F_1-Score**	**BAcc**	**Pre / Rec**	**BAcc**	**Pre / Rec**	**BAcc**	**Pre / Rec**
ConvNet	71.6	78.2	59.8	0.68	68.4	**84.4** / 52.5	59.7	**85.0** / 36.3	75.3	85.0 / 65.7
Zafar et al.	73.7	73.8	73.6	0.73	72.9	72.1 / 73.8	68.1	76.7 / 60.0	80.5	76.7 / **85.1**
Sadeghi et al.	73.6	**78.3**	68.9	0.73	71.3	74.4 / 68.9	63.7	73.3 / 54.4	76.5	73.3 / 80.6
CF-Net	**74.1**	73.4	**75.4**	**0.74**	**74.2**	73.0 / **75.4**	**69.0**	76.7 / **62.7**	**82.4**	**88.1** / 76.4

**Method**	Whole Cohort	**c**-independent subset	**c**-independent Young	**c**-independent old
**Num of Subjects**	N=357	N=244	N=115	N=129
Zafar et al.	0.213	0.105	0.072	0.661
Sadeghi et al.	0.233	0.295	0.442	0.572
CF-Net	0.069	0.035*	0.045*	0.317

* denotes significant higher prediction accuracy than ConvNet by DeLong's test (uncorrected $p < 0.05$).

variables from the features derived from each approach. For each approach, we extracted and fixed the features $\mathbf{F}$ of the control cohort in the training set, randomly initialized the confounder predictor $\mathbb{CP}$, and then (post-hoc) trained $\mathbb{CP}$ to predict the z-scored age $\mathbf{c}$ from the recorded features. The objective function of this posthoc training of $\mathbb{CP}$ was to maximize the squared correlation between predicted and ground-truth age. For each training epoch, we recorded the predicted age for the controls in the testing fold and then computed the absolute value of Pearson correlation with respect to the ground-truth age. Supplementary Figure 2a shows the average curve of Pearson correlation over the 5 testing folds, which indicates that $\mathbb{CP}$ accurately predicted age from the features learned by ConvNet resulting in a high correlation close to 1. On the other hand, the correlation associated with CF-Net remained close to 0 supporting the claim that the features learned by CF-Net were conditionally independent of the confounder variable. This observation remained valid if we replaced the squared correlation loss by the MSE loss, which was significantly lower for ConvNet. To put the MSE loss in perspective, we view the z-scores of age as samples drawn from a normal distribution and then define the theoretical MSE loss values for two 'null' classifiers. The first classifier always predicts the mean age of the cohort resulting in a theoretical loss of 1, i.e., the variance of normal distribution. The second classifier predicts the age of a randomly sampled subject so the theoretical loss is 2, i.e., the variance of the difference between two normally distributed variables. According to Supplementary Figure 2b, the features learned by ConvNet were predictive of age indicated by an MSE lower than the null losses, whereas the MSE associated with CF-Net fell in the range of null losses indicating that the features learned by CF-Net were not predictive of confounder values.

Extension of HIV Classification and Feature Space Analysis. We extended the model comparison to the loss functions of invariant-feature-learning approaches proposed by Sadeghi et al. [7] and

Zafar et al. [12]. First, Sadeghi et al. [7] proposed to use the MSE loss between the predicted and ground-truth confounder value as the adversarial loss for invariant feature learning. Their implementation was confined to the scenario where the prediction network was a logistic regression (linear classifier). To translate that method to our application, we simply replaced the correlation loss of $\mathbb{CP}$ with the MSE loss. Note, in the binary case, MSE could be replaced with the binary cross-entropy resulting in an implementation that is very similar to the one proposed in [11]. Second, the loss function of Zafar et al. [12] consisted of the loss of $\mathbb{P}$ and the magnitude of correlation between the prediction score and the confounder value. Table 1 lists the classification results from the 5-fold cross-validation and the uncorrected p-value of Delong's test associated with the accuracy improvement of each approach over the ConvNet baseline. None of the p-values met the significance threshold after Bonferroni correction ($p < 0.05/3 = 0.017$). However, based on the uncorrected threshold (two-tailed $p < 0.05$), CF-Net resulted in trend-level improvement ($p = 0.069$) in prediction accuracy over ConvNet on the whole cohort. Moreover, only CF-Net resulted in significantly higher accuracy on the $\mathbf{c}$-independent subset ($p = 0.035$) and on the younger participants from the $\mathbf{c}$-independent subset ($p = 0.045$).

Next, we investigated the impact of normal aging on the feature spaces by first training each model on the entire dataset and then computing *dcor* and MI between the age and learned features on the control group. Note, computing *dcor* and MI with respect to the features cannot be based on cross-validation as each training run will result in a different feature space. Instead, we repeated the computations 20 times by training each implementation with different random initialization. Supplementary Figure 3(a,b) shows that the metrics associated with CF-Net were significantly lower than those of other approaches (two-tailed $p < 0.001$, t-test). Taken together, these results indicate that CF-Net achieved the optimal balanced accuracy on the $\mathbf{c}$-independent subset while simultaneously demonstrating the most unbiased feature space with respect to normal aging.

Model Visualization. To compute saliency maps associated with the models, we applied the `keras-vis` visualization package [3] within a 5-fold cross-validation setting. After each training run, we com-

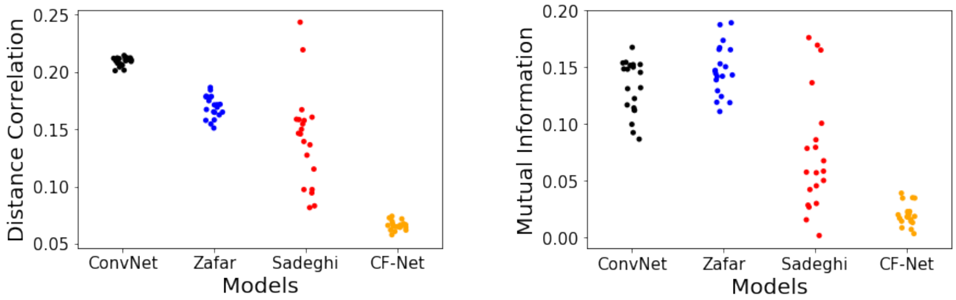

(a) (b)

Supplementary Figure 3: HIV experiment: (a) Distance correlation between the learned features $\mathbf{F}$ and subject age $\mathbf{c}$ recorded on the control subjects; (b) Mutual information between $\mathbf{F}$ and $\mathbf{c}$ recorded on the control subjects.

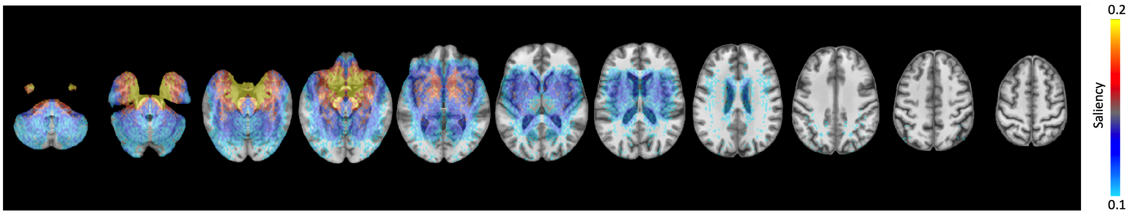

(a) ConvNet

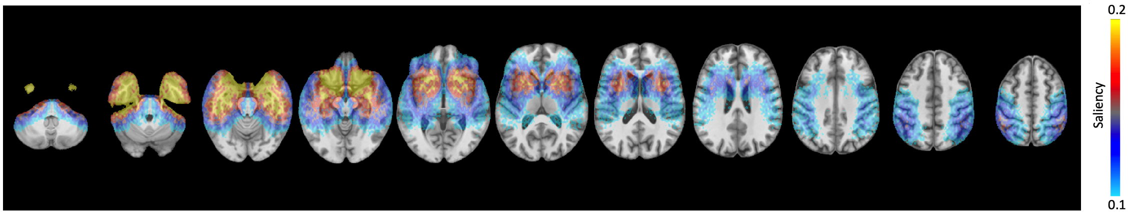

(b) CF-Net

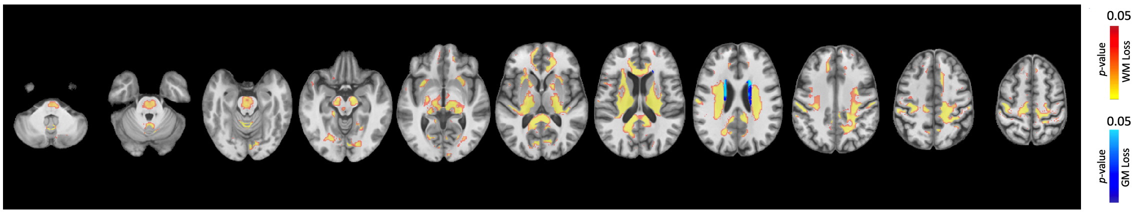

(c) VBM

Supplementary Figure 4: Brain regions associated with HIV effects identified by (a) ConvNet; (b) CF-Net; (c) Voxel-Based Morphometry.

puted the saliency map for the right hemisphere of each test image (without augmentation and flipping) based on the model learned on the training folds. We then visualize the group-level saliency map averaged over all subjects (Supplementary Figure 4(a,b). According to the SRI24 atlas [6], the top 10 regions with the highest average saliency identified by CF-Net were amygala, temporal pole (superior and middle), hippocampus, parahippocampus, orbital inferior frontal gyrus, inferior temporal gyrus, insula, olfactory, and putamen. To put these results in perspective, we identified significant tissue loss in HIV patients by conducting voxel-based morphometry analysis [5]. Based on the MR preprocessing pipeline described in the main article, tissue classification was performed by Atropos [2] resulting in Gray-Matter (GM), White-Matter (WM), and CerebroSpinal Fluid (CSF) masks for each T1w-MRI. The GM and WM masks were non-rigidly aligned to the SRI24 atlas space by registering the T1W image to a template, corrected by Jacobian determinant of the resulting deformation, and underwent Gaussian smoothing with an FWHM of 10mm. The HIV effect was tested on each voxel in the GM masks of the **c**-independent subset by a General Linear Model implemented in Permutation Analysis of Linear Models (PALM, with 5,000 permutations) [10]. Covariates of GLM included sex, age and the diagnosis label. The resulting one-tailed voxel-wise p-values associated with the diagnosis label were corrected for spatial coherence by FSL Threshold-Free Cluster Enhancement (TFCE) [8] and for family-wise error at the 5% level across space. This test procedure was then repeated on the white-matter masks. Supplementary Figure

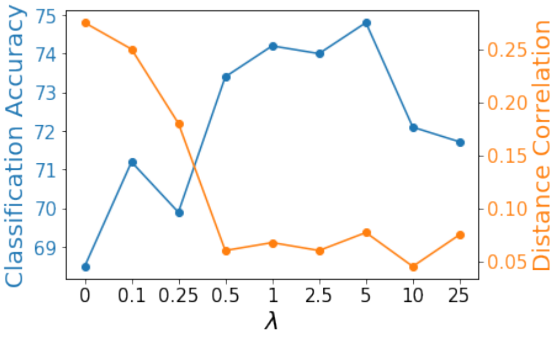

Supplementary Figure 5: The 5-fold classification accuracy and distance correlation ($dcor^2$) measures with respect to different λ in CF-Net.

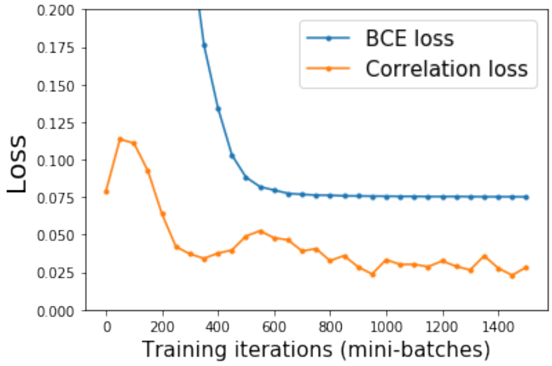

Supplementary Figure 6: Training losses of L_p and L_{cp} averaged over 5-fold cross-validation.

4c displayed voxels with significant GM (blue) and WM loss (yellow). All the aforementioned 10 regions identified by CF-Net except for the amygala showed WM loss.

Selection of λ. For each candidate $\lambda \in [0, 25]$, we performed 5-fold cross validation to record the classification accuracy on the **c**-independent subset and trained CF-Net on all subjects to measure $dcor^2$ on the control cohort. According to Supplementary Figure 5, small λ resulted in high $dcor^2$ values with age (large confounder effect) and low HIV classification accuracy. When using a large λ, CF-Net did not further reduce the $dcor^2$ metric but negatively impacted the accuracy of HIV classification as the model overemphasized the age prediction task in the feature-learning process. The range of $\lambda \in [0.5, 5]$ balanced classification accuracy with the constraint of conditional independence with respect to the confounder.

Loss Curves. Supplementary Figure 6 represents the losses of L_p and L_{cp} of CF-Net along training iterations. Both loss curves approximately converged after training with 1,000 mini-batches indicating the model simultaneously achieved accurate HIV classification (low prediction loss) and confounding effect removal (low correlation loss). The slight oscillation of L_{cp} after 1,000 iterations was likely to be the result of the competing game underlying the min-max objective (Eq. 3, main article).

Additional Results on Sex Differences in Adolescent Brains of the NCANDA Study. Similar to the HIV experiment, we inspected the statistical dependence between the learned features and the confounder by evaluating the prediction accuracy of $\mathbb{CP}$ on the features. The features of CF-Net seemed to be less predictive of PDS than those of ConvNet as they recorded lower correlations and higher MSE measures between predicted and ground-truth PDS (see Supplementary Figure 7). Unlike the HIV experiment, the MSE loss of CF-Net plotted in Fig 7b was significantly higher than that of ConvNet (($p < 0.001$ one-sample $t_{333} = 12.2$) but was marginally lower than the null losses. This indicates that the features learned by CF-Net still contained predictive information for PDS; that is, although CF-Net significantly alleviated the PDS effect in the sex prediction, it might not fully remove the effect.

Next, we extended the classification and feature space analysis with respect to the loss functions defined in Zafar et al. [12] and Sadeghi et al. [7]. For both approaches, the modeling between

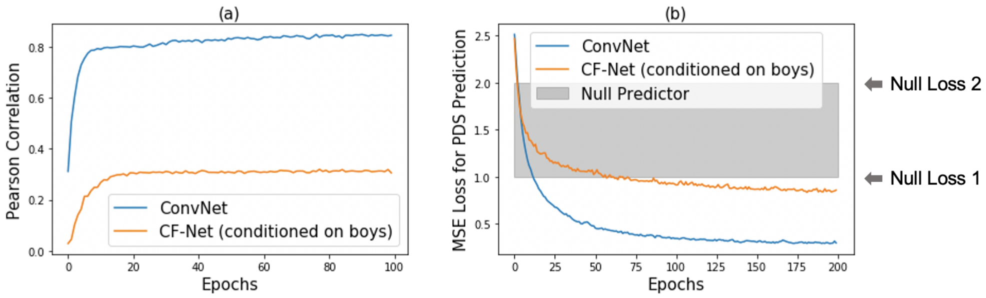

Supplementary Figure 7: NCANDA experiment: After each training run of the 5-fold cross-validation, $\mathbb{CP}$ was (re-)trained to predict PDS based on the features extracted by each implementation.

Supplementary Table 2: Top: BAcc (precision/recall) on predicting sex from MRIs of NCANDA; Bottom: Uncorrected p-value of DeLong's test associated with the improvement of each method over the Convnet baseline.

	Whole Cohort			**c**-independent		PDS<3.2		PDS>3.2	
**Method**	**BAcc**	**Pre / Rec**	**F_1-Score**	**BAcc**	**Pre / Rec**	**BAcc**	**Pre / Rec**	**BAcc**	**Pre / Rec**
ConvNet	90.3	95.5/85.2	90.5	87.3	92.5/82.5	79.5	92.8/68.1	90.6	91.0/90.0
Zafar et al.	88.6	91.4/85.6	88.4	86.7	90.5/83.0	82.6	95.6/69.5	92.9	95.6/89.8
Sadeghi et al.	86.7	92.8/80.9	86.5	82.8	90.6/75.0	79.1	89.8/69.4	85.3	92.9/77.8
CF-Net	88.8	93.6/84.1	88.6	88.5	83.8/94.0	87.8	88.4/87.0	93.0	88.4/97.0

**Method**	Whole Cohort	**c**-independent subset	PDS<3.2	PDS>3.2
**Num of Subjects**	N=674	N=400	N=138	N=262
Zafar et al.	-	-	0.667	0.533
Sadeghi et al.	-	-	-	-
CF-Net	-	0.512	0.039*	0.201

* denotes significant higher prediction accuracy than ConvNet by DeLong's test (uncorrected $p < 0.05$).
- denotes no accuracy improvement compared to ConvNet.

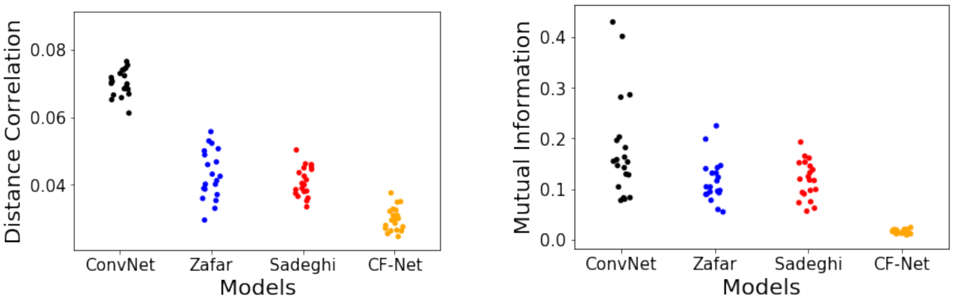

(a) (b)

Supplementary Figure 8: NCANDA experiment: (a) Distance correlation between the learned features **F** and PDS (**c**) recorded on boys; (b) Mutual information between **F** and **c** recorded on boys.

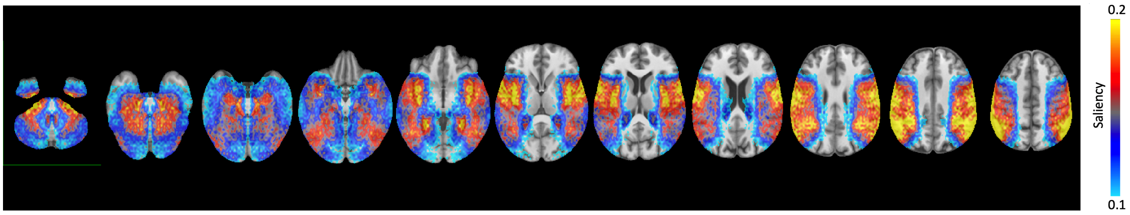

(a) ConvNet

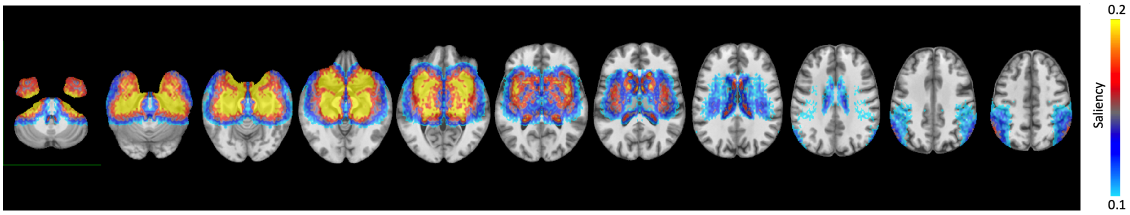

(b) CF-Net

Supplementary Figure 9: Morphological differences between the sexes of the NCANDA cohort according to ConvNet and CF-Net (conditioned on boys).

features and PDS was confined to the boys according to the analysis summarized by Table 2 (of the main article). In doing so, CF-Net achieved the highest BAcc on the **c**-independent subset and the smallest gap between the BAcc recorded for the early pubertal stage and for the late pubertal stage. Compared with the results of ConvNet, only CF-Net was significantly more accurate (two tailed $p = 0.039$, DeLong's test, Table 2) for subjects at early pubertal stage (PDS < 3.2). Meanwhile, CF-Net recorded $dcor^2$ and MI measures that were significantly lower (two-tailed $p < 0.0001$, t-test) than those reported for each of the other approaches (Supplementary Figure 8). This indicates features learned by CF-Net were more impartial to the effect of PDS.

We end the description of the results by reviewing Supplementary Figure 9, which highlights the regions with high saliency according to ConvNet and CF-Net. ConvNet relied primarily on the parietal inferior lobe, supramarginal region, cerebellum and sub-cortical regions. CF-Net, on the

other side, only focused on sub-cortical regions.

Bone Age Prediction from Hand X-Ray Images. Similar to the HIV and NCANDA experiments, we first investigated the capability of $\mathbb{CP}$ in predicting confounder values from the learned features. Since the confounder sex was a binary variable in this application, we replaced the correlation loss of $\mathbb{CP}$ with a standard binary cross-entropy (BCE). Note, this prediction task was a post-hoc analysis that did not involve adversarial training, so the standard BCE loss could directly be applied. Supplementary Figure 11 shows that the features learned by ConvNet resulted in a 76% classification accuracy of sex, which was significantly higher than the 61% achieved by CF-Net (two-tailed $p < 0.01$, DeLong's test).

Next, we extended the analysis of the bone age prediction to the feature-invariant-learning approach by Xie et al.[11], whose adversarial loss is defined by the binary cross-entropy. To enable a fair comparison, we kept all settings the same as in CF-Net other than the adversarial loss, whose training was confined to the **y**-conditioned cohort (Section 2.3, main article). According to Supplementary Figure 12b, Xie et al. [11] recorded significantly higher prediction error than CF-Net (two-tailed $p = 0.0006$, two-sample t-test), and it produced larger discrepancy between the predicted bone age in boys and girls (Supplementary Figure 12a). This sex-related discrepancy was visually confirmed in Supplementary Figure 13, which revealed the confounding effect was more pronounced in the age range of 100 months and 150 months.

References

[1] Ehsan Adeli, Qingyu Zhao, Adolf Pfefferbaum, Edith V Sullivan, Li Fei-Fei, Juan Carlos Niebles, and Kilian M Pohl. Representation learning with statistical independence to mitigate bias. *arXiv preprint arXiv:1910.03676*, 2019.

[2] Brian Avants, Nicholas Tustison, Jue Wu, Philip Cook, and James Gee. An open source multivariate framework for n-tissue segmentation with evaluation on public data. *Neuroinformatics*, 9:381–400, 03 2011.

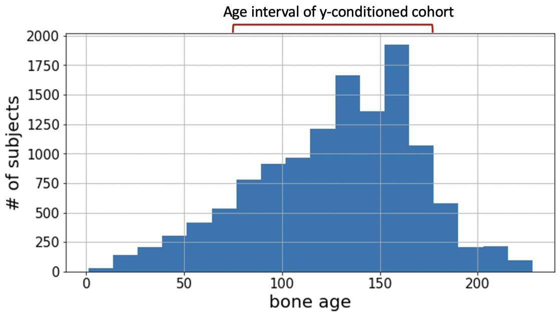

Supplementary Figure 10: Age distribution of the training subjects in the bone age experiment.

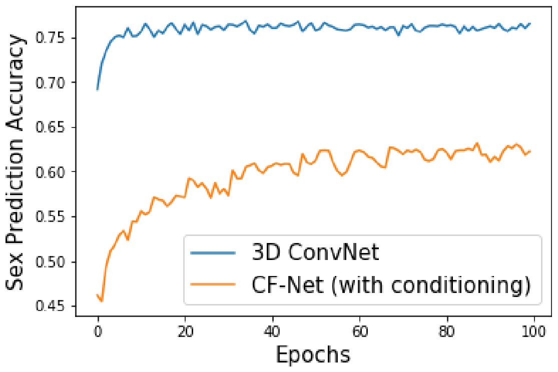

Supplementary Figure 11: Accuracy of sex prediction from the features learned by the models.

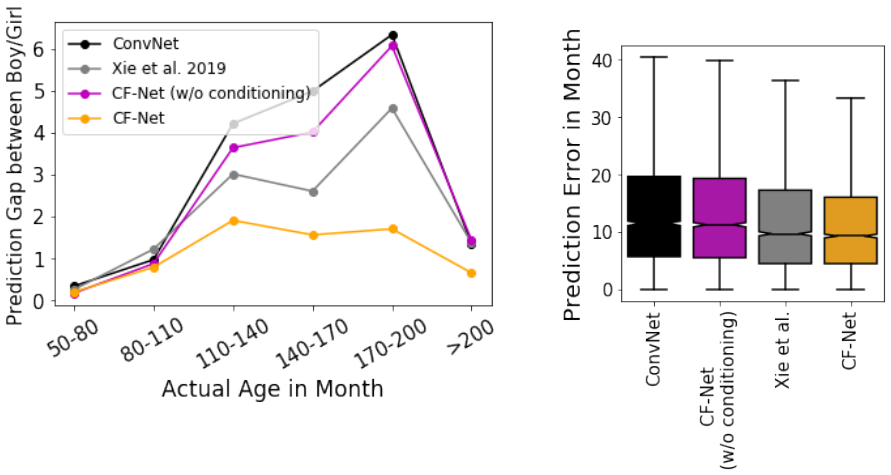

(a) (b)

Supplementary Figure 12: (a) Difference in the predicted age for boys and girls at different age spans. (b) Absolute prediction error (in months) of $n = 3,153$ testing subjects produced by different approaches. The error of CF-Net with conditioning was significantly lower than that of other approaches ($p = 0.0006$, two-tailed two-sample t-test).

[3] Raghavendra Kotikalapudi and contributors. Keras visualization toolkit. https://github.com/raghakot/keras-vis, 2017.

[4] Laurens van der Maaten and Geoffrey Hinton. Visualizing data using t-SNE. *JMLR*, 9:2579–2605, 2008.

[5] Andrea Mechelli, Cathy Price, Karl Friston, and John Ashburner. Voxel-based morphometry of the human brain: Methods and applications. *Curr Med Imaging Rev*, 1(2):105 – 113, 01 2005.

[6] Torsten Rohlfing, Natalie M Zahr, Edith V Sullivan, and Adolf Pfefferbaum. The sri24 multichannel atlas of normal adult human brain structure. *Human Brain Mapping*, 31(5):798–819, 2010.

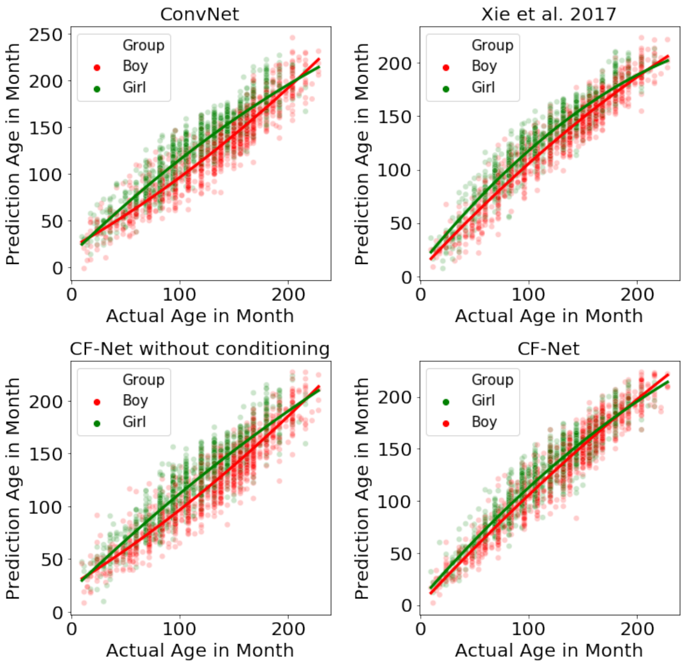

Supplementary Figure 13: Predicted age vs. ground-truth age of the 3,153 subjects in the test set of the RSNA X-ray datasets.

[7] Bashir Sadeghi, Runyi Yu, and Vishnu Boddeti. On the global optima of kernelized adversarial representation learning. In *Proceedings of the IEEE International Conference on Computer Vision*, pages 7971–7979, 2019.

[8] Stephen Smith and Thomas Nichols. Threshold-free cluster enhancement: Addressing problems of smoothing, threshold dependence and localisation in cluster inference. *NeuroImage*, 44(1):83–98, 04 2008.

[9] Gábor J Székely, Maria L Rizzo, Nail K Bakirov, et al. Measuring and testing dependence by correlation of distances. *The Annals of Statistics*, 35(6):2769–2794, 2007.

[10] Anderson Winkler, Gerard Ridgway, Matthew Webster, Stephen Smith, and Thomas Nichols. Permutation inference for the general linear model. *NeuroImage*, 92(100):381–97, 02 2014.

[11] Qizhe Xie, Zihang Dai, Yulun Du, Eduard Hovy, and Graham Neubig. Controllable invariance through adversarial feature learning. In *Advances in Neural Information Processing Systems*, pages 585–596, 2017.

[12] Muhammad Bilal Zafar, Isabel Valera, Manuel Gomez Rogriguez, and Krishna P. Gummadi. Fairness Constraints: Mechanisms for Fair Classification. In Aarti Singh and Jerry Zhu, editors, *Proceedings of the 20th International Conference on Artificial Intelligence and Statistics*, volume 54 of *Proceedings of Machine Learning Research*, pages 962–970. PMLR, 2017.
