# Supplementary material for: Training confounder-free deep learning models for medical applications
Source: Nat Commun. 2020 Nov 26;11:6010. doi: 10.1038/s41467-020-19784-9 (PMC7691500; doi:10.1038/s41467-020-19784-9)
Supplement: Supplementary file 2 — Reporting Summary [file 41467_2020_19784_MOESM2_ESM.pdf]

nature research

Corresponding author(s): Kilian M Pohl

Last updated by author(s): Oct 24, 2020

# Reporting Summary

Nature Research wishes to improve the reproducibility of the work that we publish. This form provides structure for consistency and transparency in reporting. For further information on Nature Research policies, see our Editorial Policies and the Editorial Policy Checklist.

## Statistics

For all statistical analyses, confirm that the following items are present in the figure legend, table legend, main text, or Methods section.

| n/a | Confirmed | |
|---|---|---|
| [ ] | [x] | The exact sample size (*n*) for each experimental group/condition, given as a discrete number and unit of measurement |
| [ ] | [x] | A statement on whether measurements were taken from distinct samples or whether the same sample was measured repeatedly |
| [ ] | [x] | The statistical test(s) used AND whether they are one- or two-sided<br>*Only common tests should be described solely by name; describe more complex techniques in the Methods section.* |
| [ ] | [x] | A description of all covariates tested |
| [ ] | [x] | A description of any assumptions or corrections, such as tests of normality and adjustment for multiple comparisons |
| [ ] | [x] | A full description of the statistical parameters including central tendency (e.g. means) or other basic estimates (e.g. regression coefficient) AND variation (e.g. standard deviation) or associated estimates of uncertainty (e.g. confidence intervals) |
| [ ] | [x] | For null hypothesis testing, the test statistic (e.g. *F*, *t*, *r*) with confidence intervals, effect sizes, degrees of freedom and *P* value noted<br>*Give P values as exact values whenever suitable.* |
| [x] | [ ] | For Bayesian analysis, information on the choice of priors and Markov chain Monte Carlo settings |
| [x] | [ ] | For hierarchical and complex designs, identification of the appropriate level for tests and full reporting of outcomes |
| [ ] | [x] | Estimates of effect sizes (e.g. Cohen's *d*, Pearson's *r*), indicating how they were calculated |

*Our web collection on statistics for biologists contains articles on many of the points above.*

## Software and code

Policy information about availability of computer code

| | |
|---|---|
| Data collection | No software was used for data collection. |
| Data analysis | No commercial software was used for processing the data. We used open-source software (Freesurfer) for preprocessing described in section 4.1. We implemented the data analysis and machine learning pipelines using Python. Our implementation codes of the proposed methods in the paper are released freely for public use https://github.com/qingyuzhao/br-net/ (DOI: 10.5281/zenodo.4122448). Additional preprocessing scripts can be accessed upon request. |

For manuscripts utilizing custom algorithms or software that are central to the research but not yet described in published literature, software must be made available to editors and reviewers. We strongly encourage code deposition in a community repository (e.g. GitHub). See the Nature Research guidelines for submitting code & software for further information.

## Data

Policy information about availability of data

All manuscripts must include a data availability statement. This statement should provide the following information, where applicable:

- Accession codes, unique identifiers, or web links for publicly available datasets
- A list of figures that have associated raw data
- A description of any restrictions on data availability

All data used in this manuscript are descried and their respective references are cited in the 'Material' Subsection of the 'Methods' Section. For the HIV dataset, as previously described in our previous publications, patients were recruited by referral from local outpatient HIV/AIDS treatment centers, presentations by project staff, and distribution of fliers at community events. Control participants were recruited by referral from patient participants, Internet posting, fliers, and word of mouth. Due to the Institutional Review Board constraints, this dataset is not accessible by the public. The NCANDA data used here are from the data release NCANDA_PUBLIC_BASE_STRUCTURAL_V01 (digital object identifier 10.7303/syn11541569) distributed to the public according to the NCANDA Data Distribution

agreement https://www.niaaa.nih.gov/research/major-initiatives/national-consortium-alcohol-and-neurodevelopment-adolescence/ncanda-data. The Bone Age dataset is publicly available. It is released by the Radiological Society of North America (RSNA) Radiology Informatics Committee (RIC) as a machine learning challenge for predicting pediatric bone age.

# Field-specific reporting

Please select the one below that is the best fit for your research. If you are not sure, read the appropriate sections before making your selection.

[x] Life sciences [ ] Behavioural & social sciences [ ] Ecological, evolutionary & environmental sciences

For a reference copy of the document with all sections, see nature.com/documents/nr-reporting-summary-flat.pdf

# Life sciences study design

All studies must disclose on these points even when the disclosure is negative.

| | |
|---|---|
| Sample size | We used multiple datasets in this study. Sample size was different for different each experiment. HIV: 345, NCANDA: 674; Bone Age: 14,236. In addition to the proof-of-concept (synthetic) study, we chose the 3 real datasets to cover all scenarios commonly encountered in medical applications, including 2D (CT) and 3D (MRI) modalities, classification (HIV, NCANDA) and regression (bone age) tasks, and all three types of confounder variables (age is continuous, Pubertal Development Score or PDS is ordinal, and sex is binary). |
| Data exclusions | No data was excluded from analysis. We used all data that passed the preprocessing pipelines. |
| Replication | We used 5-fold cross-validation to test the models, with multiple random splits. Cross-validation has shown to produce the most reproducible results when using machine learning models. |
| Randomization | We did not perform randomized trial. Therefore it is not relevant to our study. However, with respect to the random run of the algorithms to produce the results. All runs are with a random seeds. Our results are generated by the average of 5 runs of 5-fold cross-validation. |
| Blinding | The investigators were blinded to group allocation. Data was collected outside this study. Since we did not perform randomized trials, blinding is not relevant. We reused datasets introduced and analyzed in other manuscripts. Investigators in this paper were completely blinded to the subject IDs and group allocations during the data collection period. |

# Reporting for specific materials, systems and methods

We require information from authors about some types of materials, experimental systems and methods used in many studies. Here, indicate whether each material, system or method listed is relevant to your study. If you are not sure if a list item applies to your research, read the appropriate section before selecting a response.

## Materials & experimental systems

| n/a | Involved in the study | |
|---|---|---|
| [x] | [ ] | Antibodies |
| [x] | [ ] | Eukaryotic cell lines |
| [x] | [ ] | Palaeontology and archaeology |
| [x] | [ ] | Animals and other organisms |
| [ ] | [x] | Human research participants |
| [x] | [ ] | Clinical data |
| [x] | [ ] | Dual use research of concern |

## Methods

| n/a | Involved in the study | |
|---|---|---|
| [x] | [ ] | ChIP-seq |
| [x] | [ ] | Flow cytometry |
| [x] | [ ] | MRI-based neuroimaging |

## Human research participants

Policy information about studies involving human research participants

| | |
|---|---|
| Population characteristics | The HIV dataset contains 223 healthy controls (age 45 +/-17 years) and 122 patients (age 51 +/-8.3 years) diagnosed with HIV (CD4 count >100 ~ cells/uL). The NCANDA dataset contains 34 boys and 340 girls (age 12 to 21 years). The bone age dataset contains 6,833 boys and 5,778 girls (Boys: 134.8 +/- 42.2 months, Girls:118.7 +/-38.2 months). |
| Recruitment | No subjects were recruited by this study. All subjects were acquired by other studies references to in the manuscript. |
| Ethics oversight | The HIV study was approved by Institutional Review Board (IRB) at both SRI International (Protocol ID: Pro00039132) and Stanford University (Protocol ID: IRB-9861). Recipient acknowledges that the collection of NCANDA Data were approved by the IRB of the local collection sites in accordance with Department of Health and Human Services regulations at 45 CFR Part 46. |

Note that full information on the approval of the study protocol must also be provided in the manuscript.
